# Design and Differentiation of Quantum States at Subnanometer Scale in La_2_CuO_4_−Sr_2_CuO_4−δ_ Superlattices

**DOI:** 10.1021/acsnano.3c01422

**Published:** 2023-06-01

**Authors:** Nicolas Bonmassar, Georg Christiani, Ute Salzberger, Yi Wang, Gennady Logvenov, Y. Eren Suyolcu, Peter A. van Aken

**Affiliations:** † Max Planck Institute for Solid State Research, Heisenbergstrasse 1, Stuttgart 70569, Germany; ‡ Center for Microscopy and Analysis, Nanjing University of Aeronautics and Astronautics, Nanjing 210016, PR China; § Department of Materials Science and Engineering, Cornell University, Ithaca, New York 14853, United States

**Keywords:** superconductivity, O-K edge prepeak, oxygen vacancies, metal−insulator transition, scanning transmission electron microscopy, electron energy-loss spectroscopy, molecular beam epitaxy

## Abstract

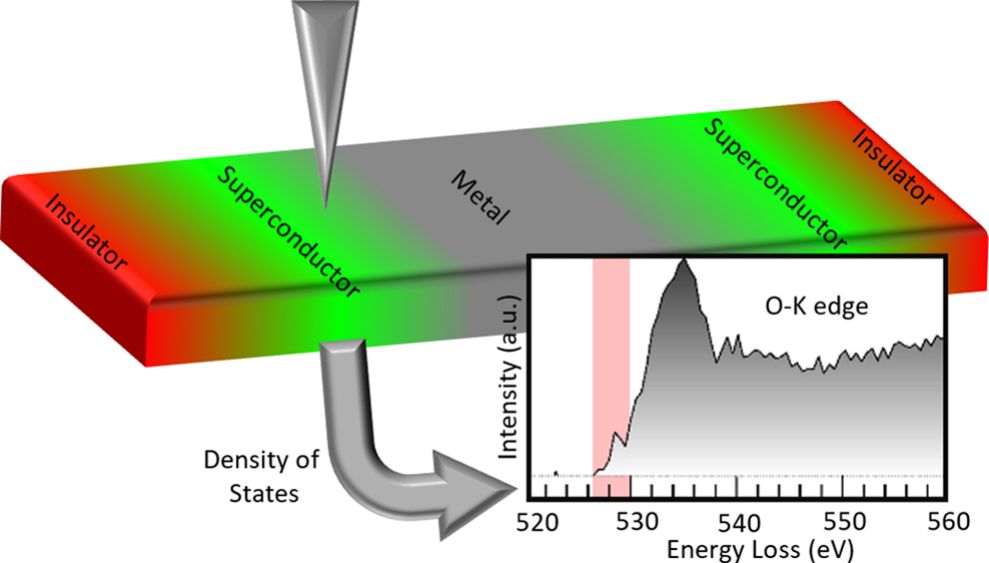

We present a study on the properties of superlattices made of ultrathin Sr_2_CuO_4−δ_ layers sandwiched between La_2_CuO_4_ layers beyond the antiferromagnetic insulating nature of the individual layers of choice. Using molecular beam epitaxy, we synthesized these superlattices and observed superconductivity and metallicity at the interfaces. We probed the hole distribution to determine the discernible quantum states and found that the high-quality epitaxy, combined with mapping the electronic fine structure by electron energy-loss spectroscopy, allowed for the differentiation of insulating, metallic, and superconducting layers at the atomic-column scale. Our results demonstrate the possibility of exploring specific electronic properties at the subnanometer scale and highlight the potential of utilizing metastable Sr_2_CuO_4−δ_ slabs.

Complex oxide materials with intricate electronic phase diagrams hold immense potential for investigating exciting physical phenomena, such as giant magnetoresistance^1,2^ and high-temperature superconductivity.^3^ La_2_CuO_4_ (LCO) serves as a model system for high-temperature superconductivity, transitioning from an antiferromagnetic insulator to a superconductor (SC) through hole^4−8^ or electron doping.^9^ The use of molecular beam epitaxy (MBE) enables the fabrication of high-quality oxide heterostructures, allowing for the engineering of physical properties at interfaces.^10,11^ For instance, in heterostructures made of two nonsuperconducting LCO layers, i.e., undoped LCO and overdoped La_2–*x*_Sr_*x*_CuO_4_ (LSCO), the intermixing of the cations changes the electronic state of the individual materials, resulting in the emergence of interfacial superconductivity.^12−14^

Characterizing the electronic states in superconducting cuprates is frequently accomplished using X-ray absorption spectroscopy (XAS)^15,16^ and electron energy-loss spectroscopy (EELS) in conjunction with scanning transmission electron microscopy (STEM).^17,18^ The prepeak of the O-K edge, which plays a key role in analyzing hole doping in superconductors,^17−19^ is the focus of most studies. In the literature, various terminology has been proposed to describe the O-K edge prepeak in LSCO. In our work, we will address this emerging O-K edge prepeak as hole peak (HP). Chemical doping, such as Sr doping in LCO, shifts spectral weight from the upper Hubbard band (UHB) to the HP, thereby introducing holes into the system.^20−22^ Polarization-dependent XAS provides information on orbital occupation through high energy resolution,^14,22,23^ but with limited spatial resolution, which is inevitable for characterizing innovative applications like Josephson junctions.^24^ EELS bridges this gap, offering atomic-scale characterization of orbital occupation, and the ability to quantify cations and anions, and their physical properties as a function of position by probing different phases.

In this work, we aimed to differentiate between metallic, insulating and superconducting phases at the interface (IF), by probing the holes and atomic distribution. To achieve this, we designed a superlattice (SL) consisting of epitaxial ultrathin tetragonal Sr_2_CuO_4−δ_ (SCO) layers sandwiched between LCO layers, using an oxide MBE system. In bulk, LCO crystallizes in a tetragonal K_2_MgF_4_-type structure, while Sr_2_CuO_3_ is orthorhombic and consists of 1D chains of CuO_4_-planes.^23^ By applying high oxygen pressure, the phase transition from orthorhombic Sr_2_CuO_3_ to the tetragonal SCO is induced.^24^ We utilize ultrathin SCO layers resulting in improved interface control when the SCO layers are sandwiched between LCO layers. This design simultaneously exhibits superconductivity, metallicity, and insulating phases and enables the analysis of holes and doping contents through STEM EELS analysis of the O-K, Cu-L_2,3_, La-M_4,5_, and Sr-L_2,3_ edges. The evolution of the O-K edge prepeak reveals the transfer of spectral weight from the UHB to the HP, which drives superconductivity. The excitations of holes resulting in HPs, the presence of oxygen vacancies, and the Sr content can be directly probed in individual atomic layers, enabling the differentiation and characterization of quantum states at the atomic scale.

## Results and Discussion

### Tuned Spectral Weight Transfer from UHB to HP

**Figure 1:**
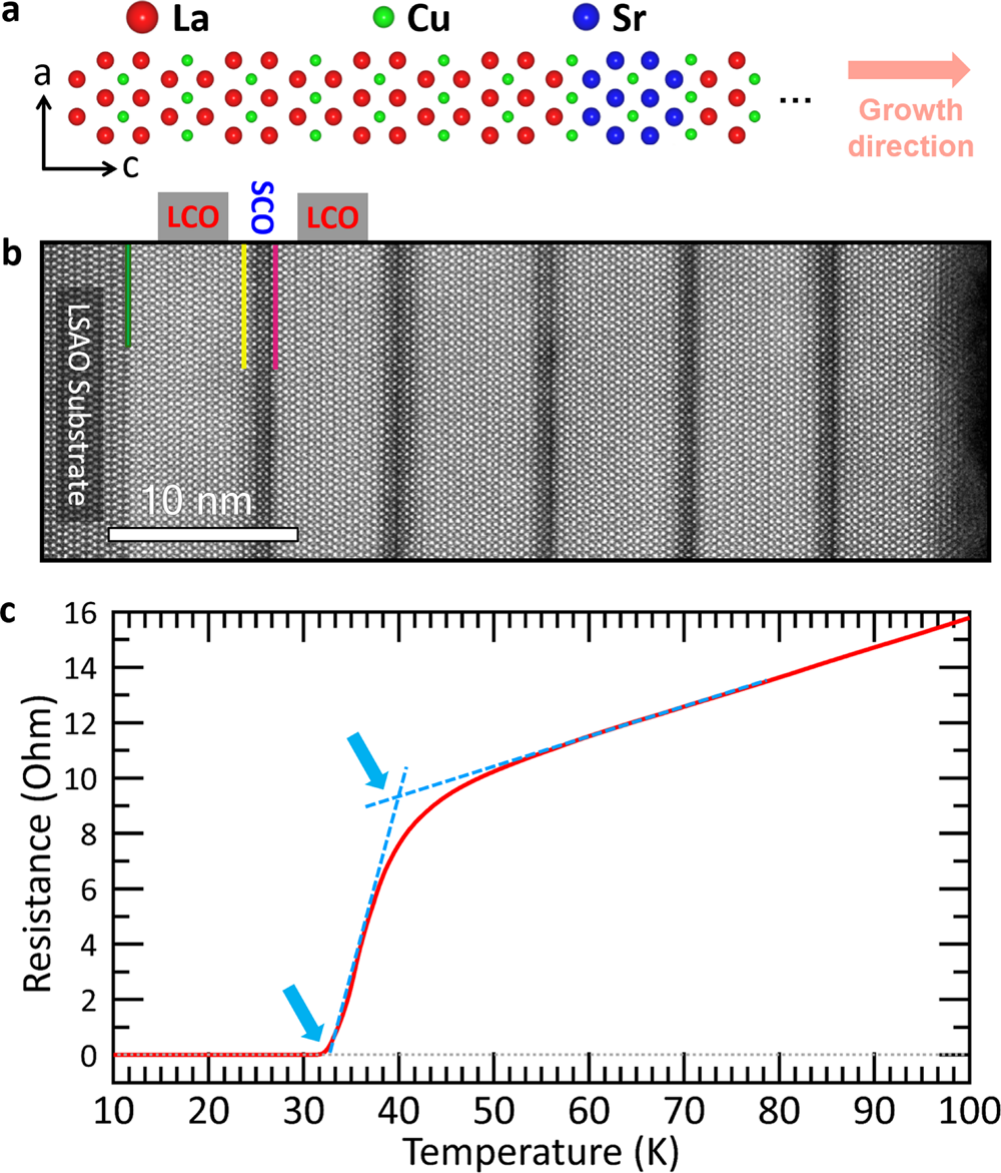
Atomically resolved STEM imaging and transport properties of the LCO-SCO SL. (a) Structural illustration of one of the five LCO-SCO blocks. Red, blue, and green represent La, Cu, and Sr, respectively. (b) ADF overview image depicting the defect-free SL consisting of a repetitive bilayer system, namely eight half unit cells LCO and two half unit cells SCO, as well as an LCO protection layer, grown on an LSAO substrate with (001) orientation along the [001] axis. The green line in panel (b) indicates the interface to the substrate. The yellow and pink arrows point out the LCO-SCO and SCO-LCO interfaces IF1 and IF2, respectively. (c) Resistance versus temperature (red) curve. The blue arrows point out the *T*_c_ onset and *T*_*R*=0_ values.

SLs composing of five repetitive LCO-SCO bilayers were grown by oxide MBE in a layer-by-layer regime and *in situ* monitored by reflection high-energy electron diffraction (RHEED). To ensure an oxygen-interstitial-free sample, all SLs were vacuum-annealed postgrowth. The structural model of the building block, consisting of LCO and SCO, is illustrated in Figure 1a. A low-magnification annular dark-field (ADF) image, shown in Figure 1b, provides a comprehensive overview of the SL grown on a LaSrAlO_4_ (001) substrate and displays a high-quality, perfect epitaxial structure without any undesired defects (see also SI Figure 1). The LCO layers appear brighter compared to the SCO layers due to the difference in atomic-weight between La and Sr (Z-contrast),^25^ while minor contrast variations are observed in the SCO layers. The chemical potential and crystallographic differences between orthorhombic Sr_2_CuO_3_ and tetragonal LCO can result in La/Sr intermixing,^26^ as discussed later (cf. Figure 3). The transport properties of this heterostructure were measured with resistance yielding a *T*_c, Onset_ of 40 K and a *T*_*R*=0_ of 32 K shown in Figure 1c, for mutual inductance measurement see SI Figure 2.

**Figure 2:**
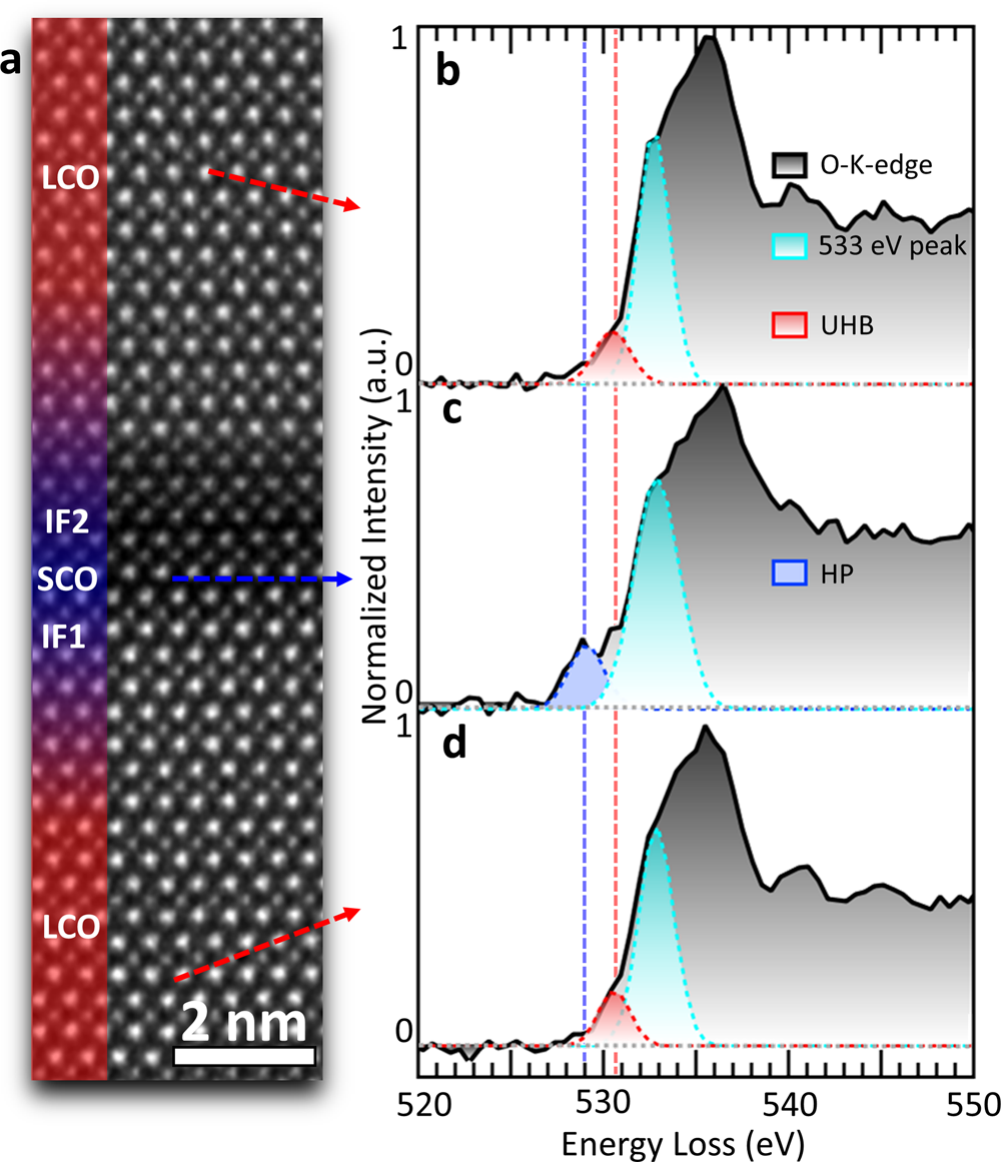
Spectral-weight transfer from the UHB to HP. (a) ADF overview of the three regions separated by two interfaces (IF1 and IF2) highlighting areas with no Sr (red) and high Sr content (blue). (b), (c), and (d) Gaussian fits with energy constraints for the HP (blue) at 529 eV, the UHB (red) at 530 eV, and a peak at 533 eV (turquoise). Spectra have been obtained from the top, middle, and bottom areas away from the interface, as indicated by the red and blue arrows.

Figure 2a presents a high-magnification image capturing the two key interfaces (IF), i.e., IF1 for LCO-SCO and IF2 for SCO-LCO. Our aim was to shed light on the transfer of spectral weight from the insulating LCO layers (UHB) to the hole-containing superconducting and metallic LSCO layers. By focusing on the prepeaks of the O-K edge (Figure 2b–d), we were able to uncover insights into this complex process. In Figure 2a, the interfacial region is further analyzed by dividing it into three different areas. An LCO area (top, Figure 2b) separated from the SCO layer (middle, Figure 2c), and an LCO area succeeding the SCO layer (bottom, Figure 2d). Using Gaussian fitting, we identified three distinct peaks, each with its own characteristics. The first peak, (*i*) the HP at ∼529 eV (blue), is situated at lower binding energies compared to (*ii*) the UHB peak at ∼530 eV (red), and (*iii*) a shoulder of the main edge onset at 533.0 eV (turquoise). The results of this analysis are visually compelling and illustrate the efficiency of spectral-weight transfer from the UHB to the HP, which is located at lower binding energies.^20,27,28^ In subsequent analyses, we aim to further dissect the interfacial area and examine the evolution of holes, Sr content, and oxygen-vacancy formation at the atomic scale.

### Probing the Interfaces: Insulating, Metallic, and Superconducting CuO Planes

**Figure 3:**
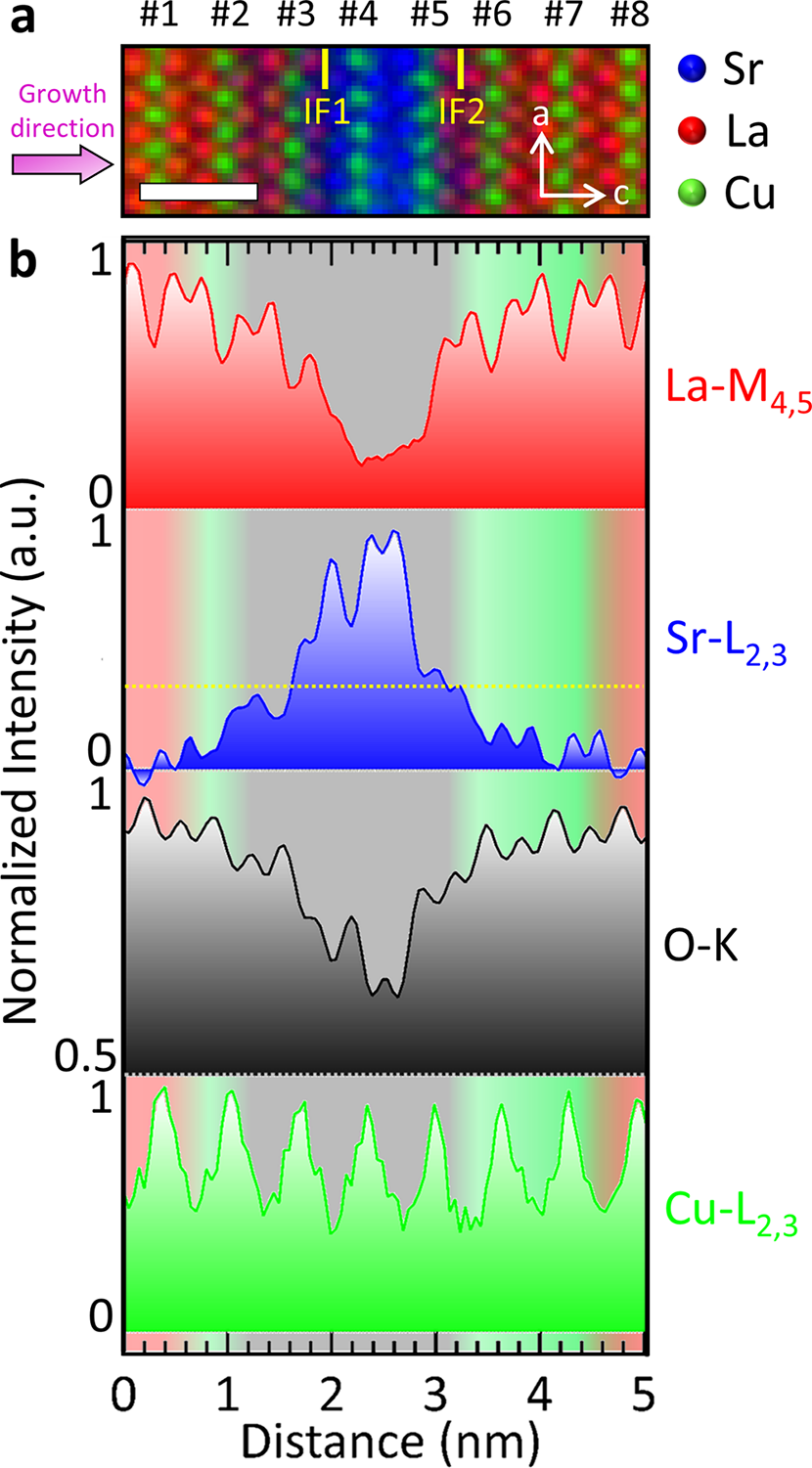
EELS spectrum imaging and corresponding elemental profiles across both interfaces, IF1 and IF2. (a) Color-coded RGB elemental map (La: red, Cu: green, and Sr: blue) of an interfacial region in the SL. The growth direction (purple arrow) was along the crystallographic c direction, and interfaces IF1 and IF2 are depicted as yellow lines. (b) Projected – La, Sr, O, and Cu profiles of the whole area at and near the interfaces. The dashed yellow line mark the Sr content obtained from samples with La_1.65_Sr_0.35_CuO_4_ layers, instead of La_2_CuO_4_ layers to differentiate between superconducting and metallic layers. The green background in (b) depicts the area, where a small Sr content is accompanied by a non-detectable amount of oxygen vacancies, the gray background depicts the area, where there are many oxygen vacancies, and the red background highlights the two areas, where neither Sr nor oxygen vacancies could be detected.

**Figure 4:**
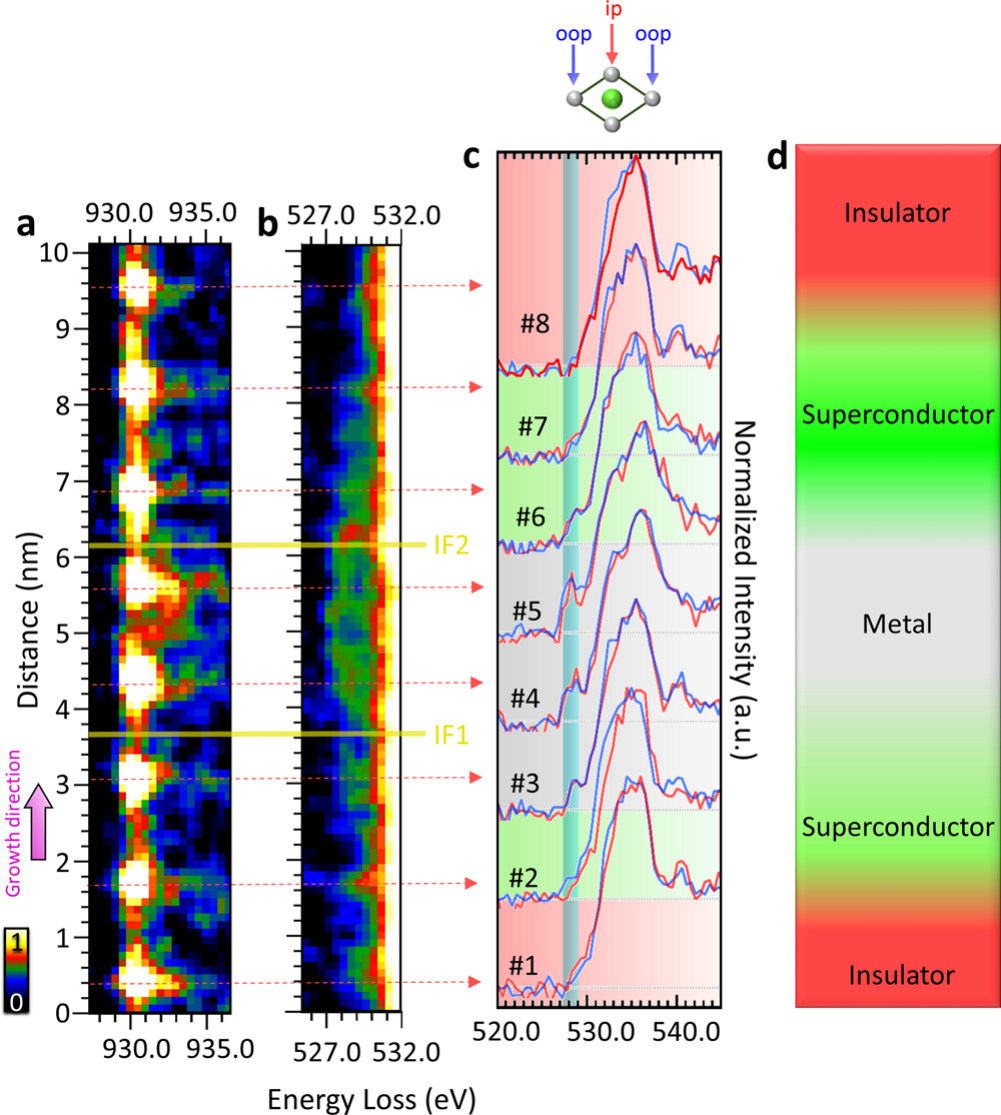
Differentiation between in-plane and out-of-plane orbital occupation via EELS spectrum imaging and O-K pre-edge analysis. Spatially resolved EELS maps of the (a) Cu-L_3_ edge and (b) O-K edge. The intensity bar in panel (a) corresponds to both heat maps, and interfaces IF1 and IF2 are depicted as yellow lines. (c) EEL spectra were collected at different positions (1–8) and subdivided into out-of-plane (blue) and in-plane (red) orbitals. The shaded region (blue) at 527–529 eV highlights the prepeak region of the O-K edge. The green background in (c) depicts the area where superconductivity occurs, the gray background depicts the area where metallicity arises, and the red background highlights the areas where an insulating phase ensues. (d) Schematic of the three distinct quantum states: insulating (red), superconducting (green), and metallic (gray) areas.

We sought to uncover the precise electronic character of the layers in question by making an initial distinction of the corresponding layer positions and correlating O-K and Cu-L_2,3_ edges. This effort is showcased in Figure 3a, where we present an atomically resolved 2D elemental map of the interfaces in detail. The eight consecutive CuO planes (green) are numbered to guide the reader through the Sr-induced holes, with green denoting the CuO planes. The first and the last Cu octahedra (planes #1 and #8) showed no detectable amount of Sr as expected for an undoped LCO layer. Conversely, Cu positions #2, #6, and #7 revealed a small Sr content but no detectable oxygen vacancies. Note that the dashed yellow line in Figure 3b highlights a Sr content of *x* = 0.35 and was obtained from EELS measurements of a SL consisting of La_1.65_Sr_0.35_CuO_4_ and Sr_2_CuO_4_. Excitingly, three Cu octahedra with high Sr content (positions #3, #4, and #5) showed evidence of oxygen vacancies (Figure 3b). With this chemical information about the elemental distribution at the interfaces in hand, we were able to correlate it with the electronic configuration regarding the hole distribution, as seen in Figure 4.

In Figure 4 a,b, the projections of the Cu-L_3_ edge and the O-K pre-edge obtained from an EELS spectrum image are shown, with the latter integrated along the crystallographic [100] direction. As for the [001] direction, spatially resolved energy-loss maps are presented. Due to the overlap of the Cu-L_3_ edge with the La-M_4_ edge, the Cu-L_3_ white lines are not used for fine structure analyses, however, are presented in the SI Figure 3. In addition, the raw spectra of La-M_4,5_ and Sr-L_2,3_ have been added in SI Figure 4. While the Cu-L_3_ edge highlights the position of the Cu columns for orientation, the O-K pre-edge region displays the in-plane (red spectra) and out-of-plane (blue spectra) orbital occupation as indicated in Figure 4c. The first Cu plane #1 with no Sr content (see black profile in Figure 3b) exhibits a shoulder indicating the UHB at 530 eV, but no holes are detected. At the second Cu position (#2), holes enter the in-plane and out-of-plane orbitals due to the presence of Sr without any detectable oxygen vacancies. With higher Sr concentrations, more holes are present, e.g., the HP increases in positions #3, #4, and #5. Between Cu position #5 and #6, a maximum of holes is depicted in the out-of-plane position, which has already been reported before in overdoped samples.^22^ This increasing HP is accompanied by an increase in oxygen vacancies, as indicated by the oxygen profile in Figure 3c (black), resulting in CuO_6_-octahedra that fulfill all requirements for metallicity, such as oxygen vacancies, high Sr content, and strong hole doping. In parallel to the second Cu position, the following two Cu positions (#6 and #7) exhibit conditions for superconductivity, including small Sr-content, holes, and no detectable oxygen vacancies. Finally, the last Cu plane (#8) shows no signs of Sr, no holes, and fully oxygenated species. Figure 4d illustrates the three distinct quantum states identified in our SL, including insulating phases (red), metallic (gray), and superconducting (green) areas.

## Conclusions

We report the successful synthesis of high-quality SCO-LCO SLs, in which distinct quantum states are generated at specific sublattices. Our findings demonstrate the delocalization of holes within the conducting layers, resulting in the establishment of three distinct quantum states: superconducting, metallic, and insulating phases. Furthermore, these three distinct states could be locally identified as insulating LCO layers and Sr-containing conducting LSCO layers.^29^ The achievement of ultrathin metallic tetragonal Sr_2_CuO_4−δ_, instead of insulating orthorhombic Sr_2_CuO_3_, is attributed to the high ozone pressure during growth and the epitaxial compressive strain induced by the LaSrAlO_4_ (001) substrate. Note that the SCO layer is metallic due to hole doping from additional oxygen in the structure, cf. additional sample presented in SI Figure 5. Based on the absence of superconductivity in strongly overdoped La_1.65_Sr_0.35_CuO_4−δ_ and SCO heterostructures (as shown in SI Figure 6), the superconductivity in SCO-LCO SLs emerges from the interfacial region and not from the SCO layer. In contrast to the absence of holes in Sr_2_CuO_3_,^30^ our SL, which shows the presence of holes (cf. Figures 2 and 4), exhibits interfacial superconductivity with *T*_*c*, onset_ ∼ 40 K (cf. Figure 1c). In an earlier study, it was demonstrated that high-temperature superconductivity emerges in a single CuO plane.^31^ Here, we show the presence of different superconducting CuO planes at the bottom and top interfaces between LCO-SCO-LCO heterostructures. The single bottom superconducting CuO plane shows a less pronounced prepeak as compared to the top superconducting CuO planes, most likely due to a broader Sr distribution in the top layers. The broad superconducting transition is a result of every superconducting CuO plane exhibiting its own *T*_c_, due to the varying distribution of Sr in the LCO matrix.^12,31^ These results demonstrate the effective control of the hole distribution and the establishment of distinct quantum states in our SCO-LCO SLs, which represents a significant advancement in the field of superconductivity.

The results of our fitting analysis (Figure 2) provide strong evidence of the atomic-scale differentiation of conducting phases in the synthesized SCO-LCO SL. Specifically, we observe two phase-pure (i.e., undoped) LCO layers separated from a Sr-doped region, where holes are detected. Our findings reveal a transfer of spectral weight from the upper Hubbard band at 530 eV in the pure insulating LCO layer to the HP at 529 eV in Sr doped LCO, where holes are present. These observations enable us to differentiate between the metallic and superconducting phases based on the number of holes and the presence of oxygen vacancies.

Our study demonstrates the versatility of our epitaxial engineering approach in enabling the transfer of spectral weight from the UHB to in-plane or out-of-plane hole states and back to the UHB in the same sample. Figure 4c showcases the distinction between the superconducting area, indicated by a green background, which indicates holes, small amounts of Sr, and no detectable oxygen vacancies, while the insulating region, highlighted by a red background, lacks holes, Sr dopants, and oxygen vacancies. With the help of STEM-EELS analyses, we provide a direct and atomic-scale visualization of the HPs through a plane-by-plane spatial mapping, with which we can distinguish between insulating (no holes), metallic (holes, oxygen vacancies, and high Sr content), and superconducting (holes, no detectable oxygen vacancies, and low Sr content) CuO-planes. Our results show the precise localization of holes in highly correlated systems with subnanometer resolution and provide a crucial tool for the characterization of heteroepitaxial^31^ or intrinsic Josephson junctions,^19,32,33^ which are attracting growing interest in oxide-based superconducting spintronics.

## Methods

### Oxide-MBE Growth

LCO-SCO bilayer systems consisting of four unit cells LCO and one unit cell SCO have been grown by atomic layer-by-layer oxide molecular beam epitaxy (oxide MBE) for five times on a LSAO (001) substrates (CRYSTAL GmbH) using ALL-MBE (DCA Instruments). The surface of the SL is protected via an additional four unit cell thick LCO capping layer. The deposition conditions during the growth were ∼1 × 10^–5^ Torr under oxidizing atmosphere consisting of ozone, radical oxygen and molecular oxygen, and 640 °C pyrometer temperature. After the SL was grown, the sample was cooled in vacuum, from 210 °C to room temperature to avoid the formation of interstitial oxygen doping as described elsewhere.^34,35^*In situ* RHEED was performed during the growth to verify the quality of each deposited atomic-layer.

### Transport Measurements and X-ray Diffraction

Resistance (*R*) measurements in four-point-probe configuration (Van der Pauw) with alternative direct currents of ±20 μA were carried out to verify superconductivity of the heterostructure. Mutual inductance (MI) measurements of the real and imaginary part of the magnetic susceptibility in a two-coil configuration (parallel geometry) with an alternative current of 50 μA and a frequency of 1000 Hz were employed. *R* and MI vs temperature (*T*) measurements were controlled by a motorized custom-designed dipstick (*T* change rate <0.1 K/s), and the temperature was varied from room temperature to 4 K (liquid helium). Out-of-plane X-ray diffraction (XRD) measurements were performed with a diffractometer equipped with a Cu-K_α_ source (Bruker D8 Cu-K_α1_ = 1.5406 Å) to check the general macroscopic quality of the sample.

### Scanning Transmission Electron Microscopy

The preparation of electron-transparent specimen included diamond-saw cutting and tripod-wedge polishing. Afterward, a precision ion polishing system (PIPS II, Model 695) equipped with a liquid nitrogen cooling stage using argon ions was employed for final thinning of all specimens with an estimated final thickness of ∼15 nm and all TEM specimens yield similar thicknesses. Scanning transmission electron microscopy (STEM) analyses have been performed with a JEOL JEM-ARM200F STEM equipped with a cold-field emission gun, a probe C_s_-corrector (DCOR, CEOS GmbH) and a Gatan GIF Quantum ERS electron energy-loss spectrometer equipped with a Gatan K2 direct electron-detection camera. EELS results and STEM images were collected at a convergence semiangle of 22 mrad resulting in a probe size of 0.8 Å. For annular dark-field (ADF) imaging, the collection-angle range was 87–209 mrad. EELS data were acquired at a collection semiangle of 87 mrad. A pixel dwell time of 7.4 ms and an energy dispersion of 0.5 eV/channel (resulting in an energy resolution of 1 eV) were used for all EELS experiments. Principle component analyses (PCA) was applied to reduce the noise in the spectrum images (SI).^36^ After PCA including 10 components, multiple linear least-squares (MLLS) fitting was performed on the PCA treated SIs as described elsewhere.^37^ For the elemental profiles, ELNES spectra and the 2D projected line scans, MLLS fitting and horizontal pixel summation were performed on raw data with no further treatment, respectively.

## Supporting Information Available

XRD θ-2θ scan, *in situ* RHEED obtained during the growth, resistance and mutual inductance vs temperature measurements, high-angle annular dark-field, annular bright-field, elemental maps and elemental profiles of an additional sample consisting of a repetitive bilayer of La_0.65_Sr_0.35_CuO_4^−^_ Sr_2_CuO_4−δ_, additional Cu-L_3_, La-M_4,5_ and Sr-L_2,3_ spectra (PDF)
