# Supplementary material for: Design and Differentiation of Quantum States at Subnanometer Scale in La2CuO4−Sr2CuO4−δ Superlattices
Source: ACS Nano. 2023 Jun 1;17(12):11521–6. doi: 10.1021/acsnano.3c01422 (PMC10311586; doi:10.1021/acsnano.3c01422)
Supplement: Supplementary file 1 — nn3c01422_si_001.pdf [file nn3c01422_si_001.pdf]

Supporting Information: Design and Differentiation of Quantum States at Sub-nanometer Scale in La_2CuO_4–$Sr_2CuO_{4-\delta}$ Superlattices

Nicolas Bonmassar[1], Georg Christiani[1], Ute Salzberger[1], Yi Wang[2], Gennady Logvenov[1], Y. Eren Suyolcu[1,3*], and Peter A. van Aken[1]*

[1] *Max Planck Institute for Solid State Research, Heisenbergstrasse 1, Stuttgart, 70569, Germany*
[2] *Center for Microscopy and Analysis, Nanjing University of Aeronautics and Astronautics, Nanjing, 210016, P.R. China*
[3] *Department of Materials Science and Engineering, Cornell University, Ithaca, NY 14853, USA.*

**to whom correspondence should be addressed*

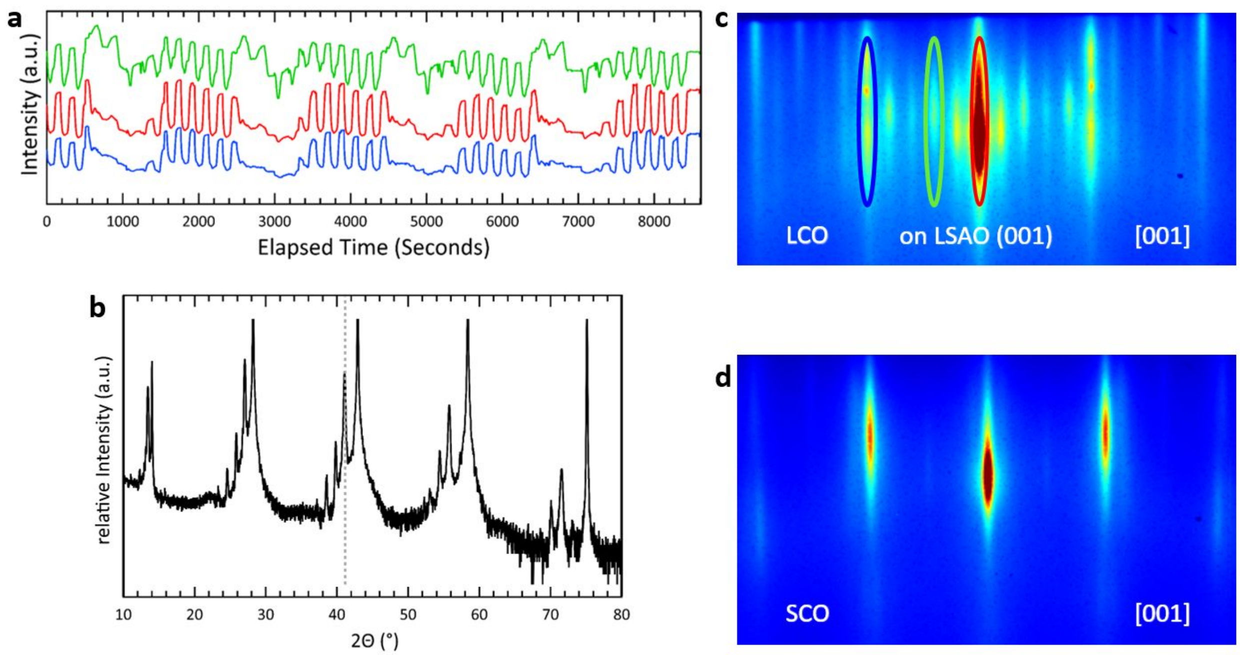

Supplementary Figure 1: *In situ*- and macroscopic structural characterizations. (a) Intensity oscillations of Bragg reflections (see (c) blue, red, and green ellipse) during the growth. (b) Out-of-plane x-ray diffraction of the superlattice with Cu-K_α source. (c) LCO and (d) SCO RHEED image, obtained during the growth.

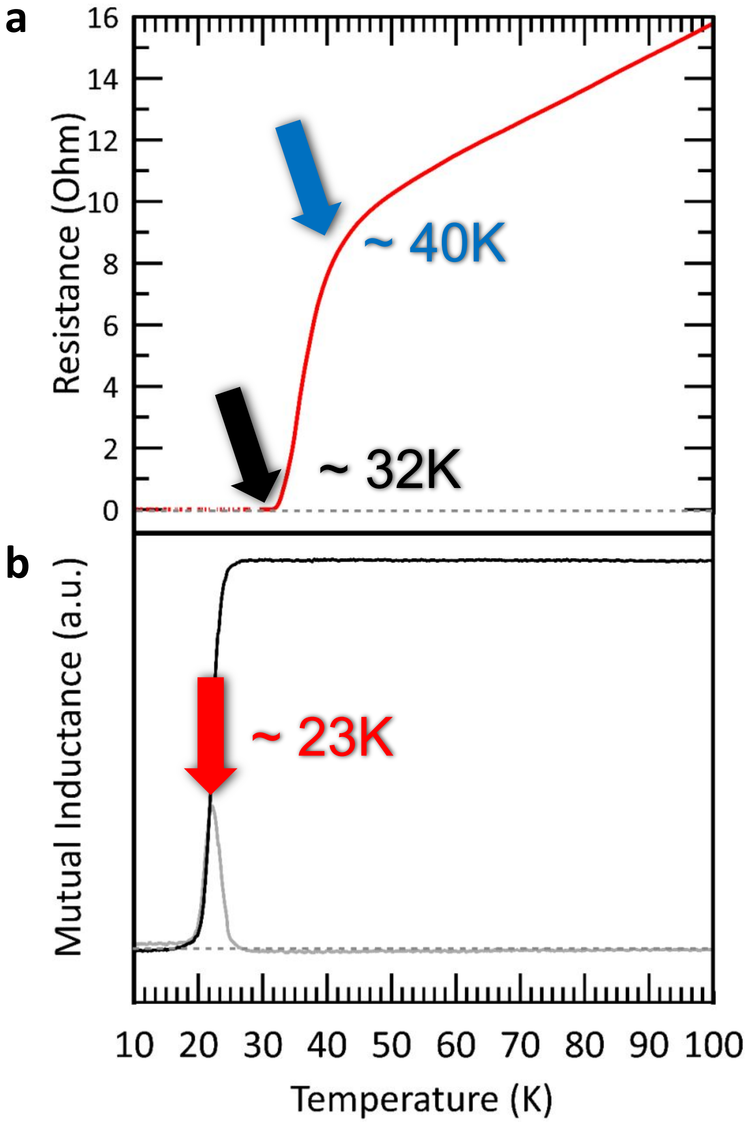

Supplementary Figure 2: Transport measurements. (a) Resistance and (b) mutual inductance vs temperature measurements. The blue and black arrows in panel (a) depict the onset and the R=0 temperatures from R vs T, respectively. The red arrow in panel (b) highlights the maximum of the imaginary part (grey line) and the inflexion point of the real part (black curve).

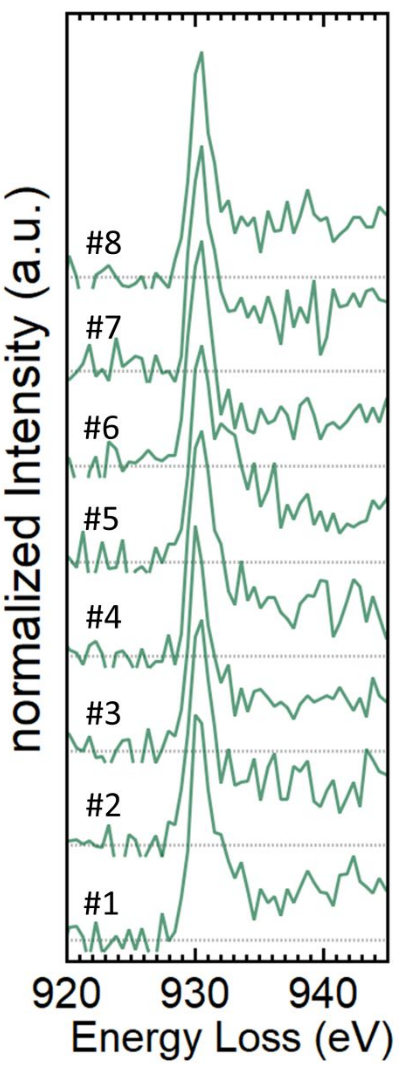

Supplementary Figure 3: Cu-L_3 edges extracted from the same location as in Figure 4c in the main text. The numbers corresponds to the same positions as in the main text.

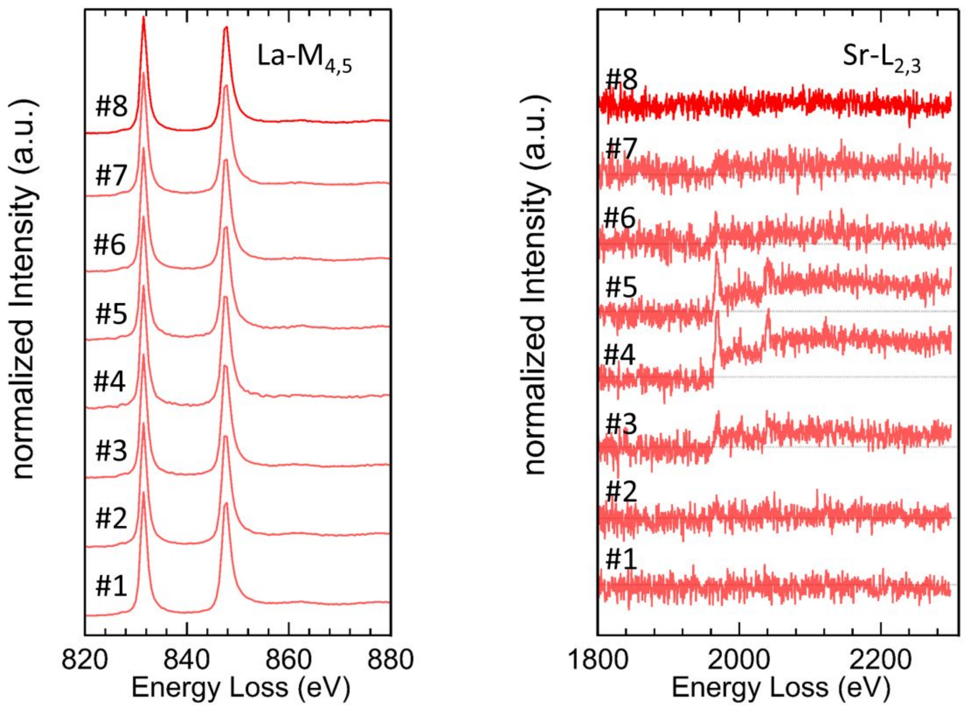

Supplementary Figure 4: La-$M_{4,5}$ and Sr-$L_{2,3}$ edges extracted from the same location as in Figure 4c in the main text. The numbers corresponds to the same positions as in the main text.

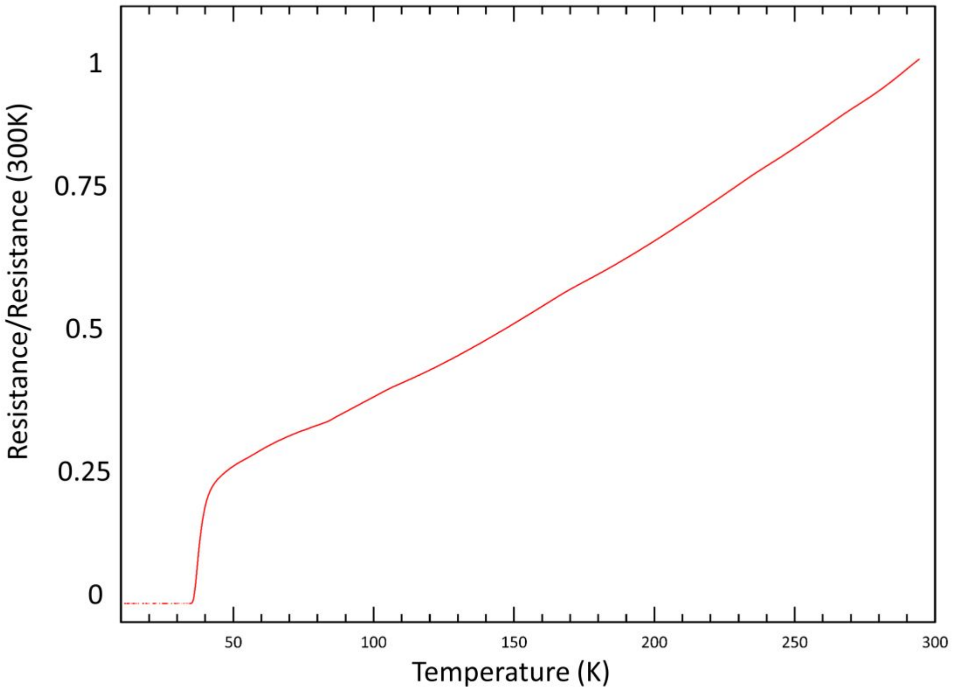

Supplementary Figure 5: Sheet resistance normalized by the resistance value at 300K versus temperature of another superlattice with thicker SCO (two unit cells) and thinner LCO (three unit cells) layers. Here, the non-linear behavior for temperatures above Tc resembles a more Fermi liquid behavior.

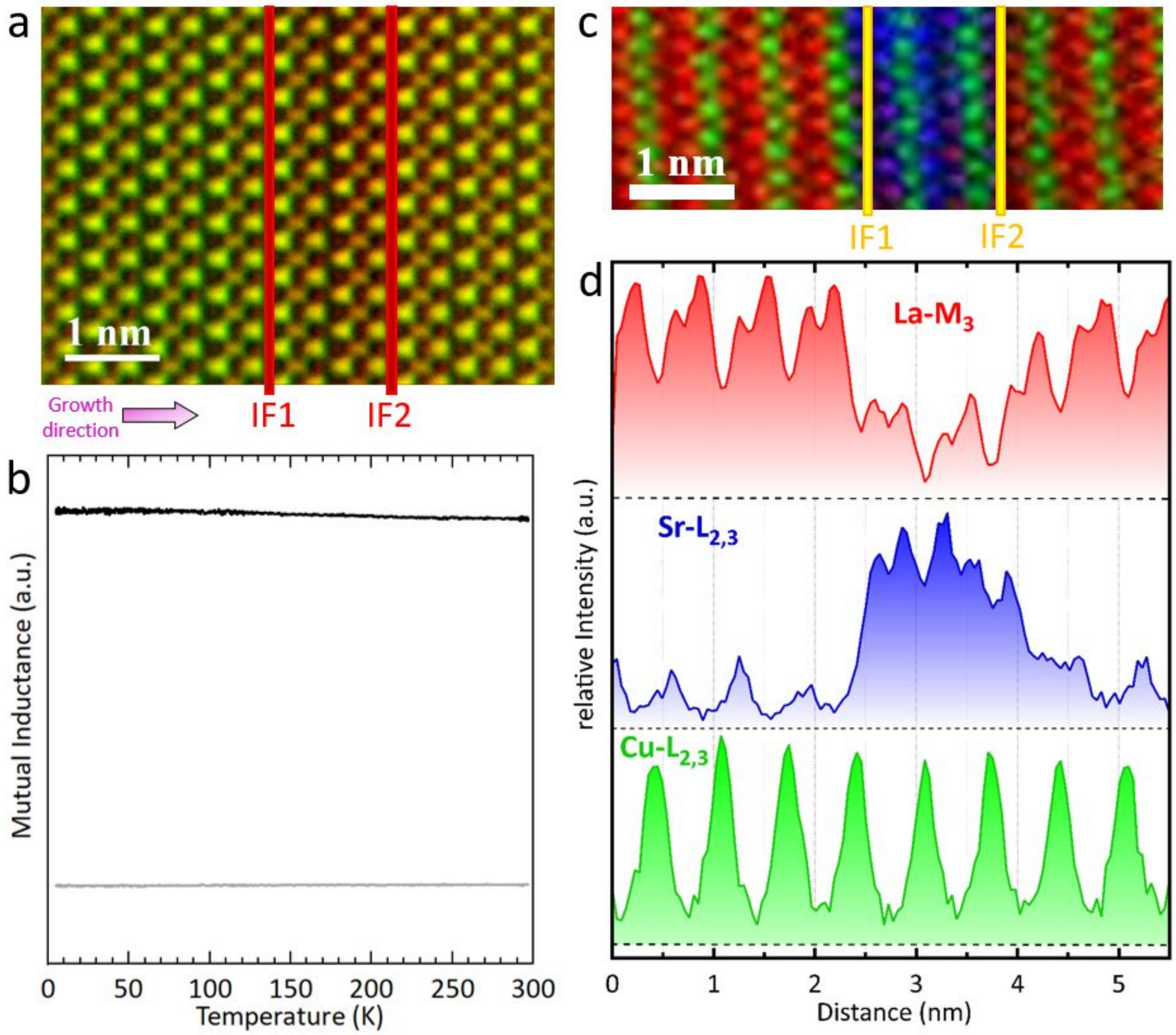

Supplementary Figure 6: Additional sample composing of $La_{0.65}Sr_{0.35}CuO_4$ -$Sr_2CuO_{4-\delta}$ -$La_{0.65}Sr_{0.35}CuO_4$. (a) Overlay of HAADF and inverted annular bright-field (iABF) images highlighting both interfaces, IF1 and IF2. (b) Real (black) and imaginary (gray) parts of mutual inductance vs. temperature measurement. (c) EELS elemental map depicting Cu (green), La (red), and Sr (blue) positions in the superlattice. (d) Profiles obtained as in [100] direction integrated elemental distribution along the [001] direction.
